# Cannabis-induced ischemic stroke involving the basilar and middle cerebral arteries in a 50-year-old man: a rare case report

**DOI:** 10.1093/omcr/omaf205

**Published:** 2025-10-29

**Authors:** Omair Bseiso, Anas Zahdeh, Wasef Alhroub, Maaweya Jabareen, Hasan I Hroob, Sharif Issa Basal, Ashraf Al-Zughayyar, Loai Muhtaseb

**Affiliations:** Faculty of Medicine, Hebron University, University Street, Hebron p720, Palestine; Faculty of Medicine, Hebron University, University Street, Hebron p720, Palestine; Faculty of Medicine, Hebron University, University Street, Hebron p720, Palestine; Faculty of Medicine, Hebron University, University Street, Hebron p720, Palestine; MD-MBBS, Internist, Faculty of Medicine, Hebron Universiy, Medical intensive care unit, Al-Ahli Hospital, Deir Sammit Street, Hebron p764, Palestine; Neurosurgeon, Intervention Neuroradiology, Hebron Street, Hebron p720, Palestine; Internist, Intensivist, Head of medical ICU / Al-Ahli Hospital, Hebron Street, Hebron p720, Palestine; Faculty of Medicine, Palestine Polytechnic university, Medical intensive care unit, Al-Ahli Hospital, Abu Kteila Street, Hebron p720, Palestine

**Keywords:** cannabis-associated stroke, large-vessel occlusion, middle cerebral artery (MCA) occlusion, basilar artery occlusion

## Abstract

Cannabis use has been increasingly associated with cerebrovascular events, though simultaneous large-vessel occlusions in both anterior and posterior circulations are rare. We report a case involving a 50-year-old male with no medical history who presented with decreased consciousness and left-sided hemiplegia. CT angiography imaging revealed acute occlusion of the right middle cerebral artery (MCA) and the basilar artery. Urine toxicology was positive for cannabis. Extensive cardiac and laboratory workups were unremarkable. The patient underwent successful mechanical thrombectomy, achieving full reperfusion (TICI 3) without complications. With no identifiable alternative cause and a history of chronic cannabis use, a diagnosis of cannabis-associated large-vessel ischemic stroke was made. To our knowledge, this is the first reported case of concurrent MCA and basilar artery occlusions in this context. This case highlights the need for increased awareness of cannabis as a potential risk factor for multi-territory ischemic stroke, particularly in patients without traditional vascular risk factors.

## Introduction

Cannabis is one of the most widely used psychoactive substances globally, with increasing legalization and social acceptance contributing to a rise in its consumption. While it is often perceived as relatively safe, growing evidence links cannabis use to a spectrum of acute and chronic health effects, including cardiovascular and cerebrovascular complications. Several case reports and observational studies have described an association between cannabis use and ischemic stroke, particularly in young and otherwise healthy individuals [1, 2].

Proposed mechanisms for drug-associated stroke include reversible cerebral vasoconstriction, cardioembolism, hypotension, and prothrombotic effects, which have been observed in association with both stimulants such as amphetamines and cocaine, as well as cannabinoids [3, 4]. Synthetic cannabinoids are believed to cause ischemic strokes through cerebrovascular effects that lead to hypoperfusion [4]. Cannabinoid receptors CB1 and CB2 have been shown to play roles in cerebrovascular regulation and neuroinflammation in experimental models of stroke, suggesting a potential involvement in the pathogenesis of cannabis-associated cerebrovascular events [5]. Reports implicate synthetic cannabinoids in stroke incidents, highlighting their potentially more toxic vascular effects [4].

## Case report

A 50-year-old male with no known history of chronic medical conditions presented to the emergency department with acute onset of decreased level of consciousness and left-sided hemiplegia. His son reported that the patient was a chronic cannabis user for approximately 20 years, typically consuming two cigarettes per day. No recent illness, trauma, or medication use was reported. The patient was intubated on arrival due to reduced consciousness, with an initial Glasgow Coma Scale (GCS) score of 10. Neurological examination revealed bilateral reactive pupils (+2) and dense hemiplegia on the left side.

Vital signs on admission were stable. Initial blood work, including complete blood count, serum electrolytes, liver and renal function tests, and electrocardiogram (ECG), were within normal limits. Laboratory investigations were unremarkable. Inflammatory markers were within normal range except for a mildly elevated C-reactive protein (CRP) titer of 18.9 mg/l and lactic acid level of 26.9 mg/dl.

Infectious screening was negative for HIV (<0.1), HCV (<0.1), and HBsAg (<0.3). Coombs direct and indirect tests were negative, and urinalysis was unremarkable. The patient’s blood type was O positive.

Urine toxicology was positive for cannabinoids and negative for all other substances, including opioids, cocaine, and amphetamines. Arterial blood gas (ABG) analysis showed respiratory alkalosis with a pH of 7.48, pCO₂ of 25.3 mmHg, pO₂ of 77 mmHg, and HCO₃^−^ of 22.0 mmol/l.

**Figure 1:**
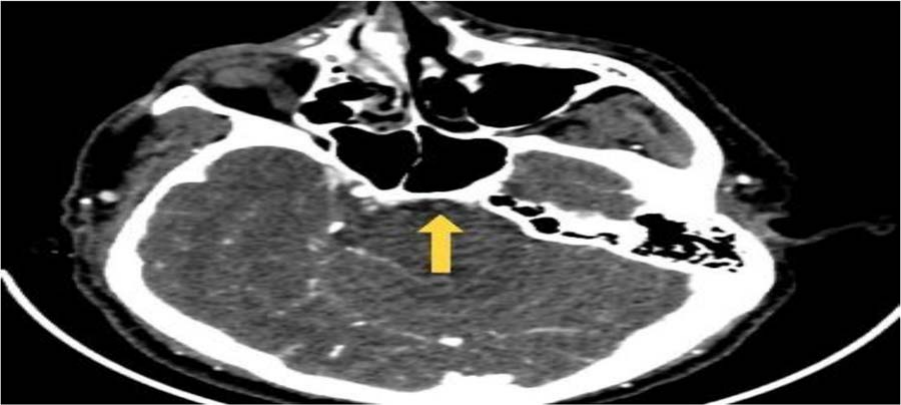
Axial CTA image of the brain demonstrating abrupt loss of contrast opacification at the proximal basilar artery (arrow), consistent with acute basilar artery occlusion.

**Figure 2:**
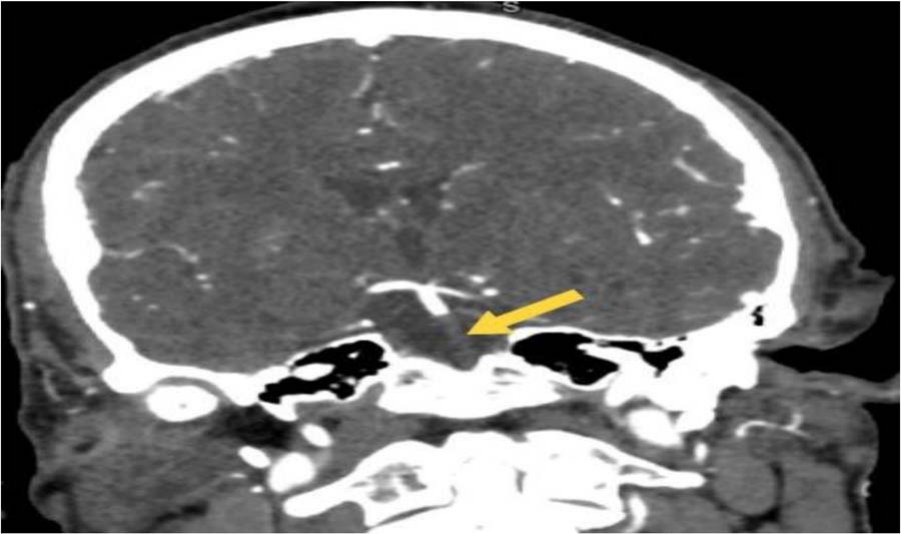
Coronal CTA image of the brain demonstrating abrupt loss of contrast opacification at the origin of the basilar artery (arrow), confirming the presence of a proximal basilar artery occlusion.

**Figure 3:**
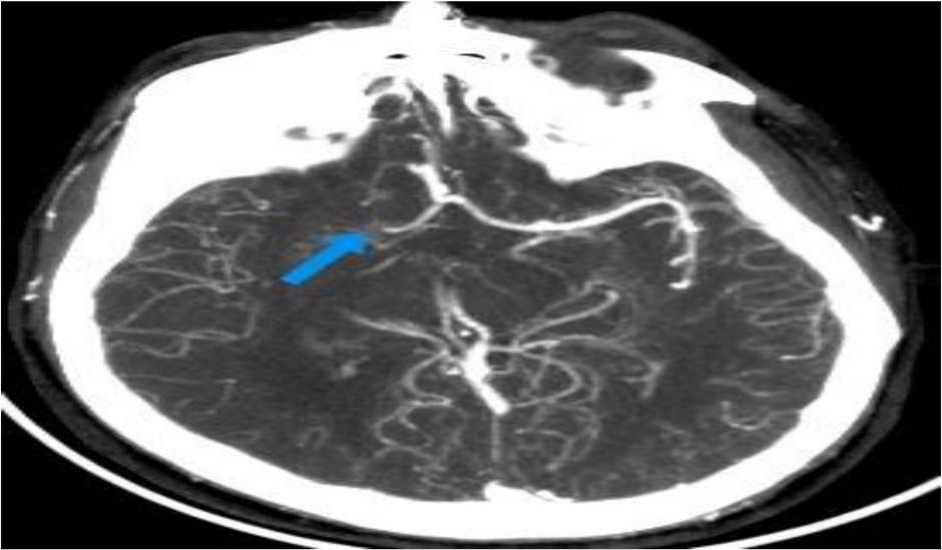
Axial CTA image of the brain showing abrupt cutoff of the right middle cerebral artery (MCA) at the M1 segment (arrow), with reduced opacification of its distal cortical branches. These findings are suggestive of a right MCA occlusion.

Non-contrast CT of the brain was unremarkable for hemorrhage. Computed tomography angiography (CTA) revealed occlusion of the basilar artery (Figs 1 and 2) and right middle cerebral artery (MCA) (Fig. 3). The patient was immediately taken for cerebral angiography, which confirmed the dual occlusions.

Mechanical thrombectomy was performed using a stent retriever and aspiration technique. Complete revascularization was achieved in both vessels, with a thrombolysis in Cerebral Infarction (TICI) score of 3. Post-procedure CT angiography imaging showed successful reperfusion with no evidence of hemorrhagic transformation. Neurological examination was unremarkable.

The patient was admitted to the intensive care unit for close monitoring and supportive care. Further workup, including echocardiography and hypercoagulability panel, was unremarkable. The clinical course and imaging findings, along with the absence of other risk factors, supported a diagnosis of cannabis-associated large-vessel occlusion.

## Discussion

Cannabis use has been increasingly implicated in the pathogenesis of ischemic stroke, particularly among younger individuals with no traditional vascular risk factors [1]. Potential mechanisms of cannabis-induced stroke include vasospasm, hypotension, cardiac arrhythmias, and vasculitis [3].

This case is particularly remarkable due to the simultaneous involvement of two major cerebral vessels—the middle cerebral artery (MCA) and the basilar artery—in a patient with a history of chronic cannabis use and no conventional vascular risk factors. Although previous reports have associated cannabis with ischemic stroke, the majority describe isolated involvement of either the anterior or posterior circulation, making concurrent large-vessel occlusion across both territories highly uncommon [6].

A case report and literature review described a 47-year-old woman in South Korea with no prior medical history who developed acute ischemic stroke after smoking cannabis. She presented with neurological symptoms including right gaze deviation and left neglect [7]. Another case involved a 42-year-old chronic cannabis user experienced recurrent transient ischemic attacks. CT angiography imaging showed reversible narrowing of the bilateral middle cerebral arteries and left posterior cerebral artery, linked to cannabis-induced vasoconstriction [8]. To our knowledge, this is the first documented case of simultaneous basilar and middle cerebral artery occlusion temporally associated with cannabis use.

In this case, the patient had no significant medical history, was negative for other substances, and had normal cardiac and coagulation workup. The abrupt onset of symptoms, imaging findings, and positive cannabis screen suggest a possible association, especially given the absence of other etiologies. Extensive laboratory evaluation did not identify alternative causes of stroke. The patient had no signs of infection, autoimmune disease, or metabolic imbalance. Inflammatory and coagulation markers, including procalcitonin and ferritin, were within or near normal range, and infectious serologies (HIV, HCV, HBV) were negative. Thyroid function tests were unremarkable. The only notable abnormal findings were a positive urine screen for cannabis and a mild elevation in CRP and lactic acid, the latter likely secondary to acute ischemia.

Cannabis has been implicated in ischemic stroke through several interconnected pathophysiological mechanisms. One of the primary mechanisms is reversible cerebral vasoconstriction (vasospasm), frequently observed in case reports of cannabis-associated strokes. Δ9-tetrahydrocannabinol (THC), the main psychoactive component of cannabis, may activate the sympathetic nervous system, resulting in vasospasm, fluctuations in blood pressure, and endothelial dysfunction, all of which can contribute to cerebral ischemia [4, 9].

Timely recognition and intervention were critical in this case. Mechanical thrombectomy remains the gold standard for large-vessel occlusions and was successful in restoring full perfusion (TICI 3) in both territories. However, the severity and location of occlusions highlight the potential for catastrophic outcomes, particularly if diagnosis or intervention is delayed.

This case emphasizes the need for heightened clinical suspicion of substance-induced vascular events, especially as global cannabis use continues to rise. Clinicians should consider recent cannabis use as part of the differential diagnosis in acute stroke presentations, even in patients without classic risk factors.

## Conclusion

This case highlights a rare and severe presentation of cannabis-associated ischemic stroke involving simultaneous occlusion of the basilar and middle cerebral arteries. In the absence of traditional risk factors, recent cannabis use was the most likely precipitating factor. As cannabis becomes more widely used, clinicians should maintain a high index of suspicion for its potential role in cerebrovascular events. Prompt recognition and intervention, such as mechanical thrombectomy, are essential for optimizing outcomes in these patients.
